# Improvement in Saccharification Yield of Mixed Rumen Enzymes by Identification of Recalcitrant Cell Wall Constituents Using Enzyme Fingerprinting

**DOI:** 10.1155/2015/562952

**Published:** 2015-06-09

**Authors:** Ajay Badhan, Yu-Xi Wang, Robert Gruninger, Donald Patton, Justin Powlowski, Adrian Tsang, Tim A. McAllister

**Affiliations:** ^1^Agriculture and Agri-Food Canada, Lethbridge Research Centre, Lethbridge, AB, Canada T1H 4P4; ^2^Centre for Structural and Functional Genomics, Concordia University, Montreal, QC, Canada H4B 1R6

## Abstract

Identification of recalcitrant factors that limit digestion of forages and the development of enzymatic approaches that improve hydrolysis could play a key role in improving the efficiency of meat and milk production in ruminants. Enzyme fingerprinting of barley silage fed to heifers and total tract indigestible fibre residue (TIFR) collected from feces was used to identify cell wall components resistant to total tract digestion. Enzyme fingerprinting results identified acetyl xylan esterases as key to the enhanced ruminal digestion. FTIR analysis also suggested cross-link cell wall polymers as principal components of indigested fiber residues in feces. Based on structural information from enzymatic fingerprinting and FTIR, enzyme pretreatment to enhance glucose yield from barley straw and alfalfa hay upon exposure to mixed rumen-enzymes was developed. Prehydrolysis effects of recombinant fungal fibrolytic hydrolases were analyzed using microassay in combination with statistical experimental design. Recombinant hemicellulases and auxiliary enzymes initiated degradation of plant structural polysaccharides upon application and improved the *in vitro* saccharification of alfalfa and barley straw by mixed rumen enzymes. The validation results showed that microassay in combination with statistical experimental design can be successfully used to predict effective enzyme pretreatments that can enhance plant cell wall digestion by mixed rumen enzymes.

## 1. Introduction

Rising grain prices heightened concerns over the use of food as feed for livestock production and the negative impacts of annual crops on carbon sequestration and biodiversity has prompted research into finding ways to increase the use of fibrous forage in ruminant diets. Plant cell walls can constitute a primary source of nutritional energy for ruminants. However for many types of forage, less than 50% of the cell wall fraction is digested and utilized by the ruminant host [1]. Substantial benefits would be realized if a greater percentage of this potential energy was made available for fermentation in the rumen through an increase in the digestibility of the cell wall fraction.

Fiber digestion in ruminants occurs primarily in the rumen and cecum. Generally, the amount of fiber digested in the lower tract is relatively small, with the rumen being the primary site of digestion. The ruminal microbial population secrets diverse hydrolases to degrade and ferment structural carbohydrates in plant cell walls. The physical and chemical nature of forages can present a barrier to their complete digestion in the rumen [2]. Therefore, prior knowledge about the structural aspects of cell wall polymers that limit digestion is critical to identifying efficient enzymatic pretreatments. In this study enzyme fingerprinting was used in combination with Flourier infrared spectroscopy (FTIR) to identify recalcitrant factors that limit fiber digestion by mixed rumen enzymes.

Exogenous enzymes have been used to remove antinutritional factors from feeds, to increase the digestibility of existing nutrients, and to supplement the activity of the endogenous enzymes. To date, they have primarily been used in poultry and swine production [3, 4]. In cattle, the addition of cellulases and xylanases directly to feeds has been shown to increase the* in vivo* numbers of fibrolytic rumen bacteria that utilize the secondary products of cellulose digestion [5]. Feedstuffs are structurally complex; each substrate presents its own set of recalcitrant components that limit the extent of feed digestion in the rumen. Ultimately, enzyme pretreatments should be designed specifically to overcome the constraints limiting digestion of different types of forages. There have been number of reports recently focusing on development of synthetic formulation of lignocellulolytic enzymes and chemical pretreatments for biomass use in biofuel production. Synthetic enzyme mixtures for ammonia fiber expansion (AFEX) treated corn stover deconstruction have been reported [6, 7]. Similarly optimized synthetic mixture of enzymes from* Trichoderma reesei* for hydrolysis of steam exploded wheat straw [8] and enzyme formulations to enhance performance of commercial enzymes against alkaline pretreated barley straw and alfalfa have been recently reported [9].

In this study we sought to use a microassay procedure in combination with statistical experimental design to predict the optimized synergy between enzyme prehydrolysis and maximum solubilization of cellulose by mixed rumen enzymes (rumen endogenous enzymes). The optimized enzyme pretreatment conditions were then validated using a scale-up assay. A similar approach using a combination of statistical design and microplate technique for enzymatic hydrolysis with comparable protein to biomass load and reaction volumes has been reported previously [9, 10]. The present work describes the use of a technique to specifically assay very small quantities of enzymes, enabling the screening of a large number of recombinant enzymes from novel sources for their ability to enhance the digestion of plant cell walls by mixed rumen enzymes. It is expected that the method described here will facilitate the development of enzyme cocktails for use as ruminant feed additives.

## 2. Material and Method

### 2.1. Source of Enzymes, Production, and Biochemical Characterization of Recombinant Enzymes

The source of recombinant enzymes, their expression, biochemical characterization (Table 1), along with details about commercial enzymes, and the methods used to prepare mixed rumen enzymes have been previously reported [9].

### 2.2. Statistical Design

Simplex-lattice designs were created using Design-Expert software (Version 8.0; Stat-Ease, Inc., Minneapolis, MN; http://www.statease.com) as described earlier [9] with slight modifications. Details of augmented special quadratic design for experiment number 1 (six components) and experiment number 2 (seven components) with 28 and 41 separate assays are shown in Tables 2, 3, 4, and 5, respectively. The relative abundance of core enzymes (i.e., Accellerase 1500 and Accellerase XC) was set to vary from 25% to 75%, while upper and lower limits for fungal enzymes were set between 50% and 100% in assay mixtures for experiment 1, whereas, in order to optimize the efficiency of enzymatic prehydrolysis, upper and lower limits for all enzymes were set to vary from 100% to 0% in experiment 2.

### 2.3. Experimental Detail

#### 2.3.1. Experiment Number 1: Enzyme Fingerprinting of Alkaline Peroxide Pretreated (AP) Barley Silage and Total Tract Indigested Fiber Residues (TIFR)

Enzymatic fingerprinting of AP treated barley silage and total tract indigestible fiber residue (TIFR) was used in this study to gain insight into the recalcitrant components in plant cell walls that may respond to enzyme pretreatment and enhance the activity of mixed rumen enzymes. Alkaline peroxide treatment was used for selective delignification of cell walls in order to enable enzymes to access inner core cellulose and hemicellulose which would otherwise have remained inaccessible.

*(a) Alkaline Peroxide Pretreatment of Barley Silage and TIFR*. Heifers (five) were fed a barley silage-based diet (70 : 30 barley silage to barley grain) with approximately 65% of dietary neutral detergent fiber coming from barley silage, as described earlier [11]. Samples (*n* = 5) of barley silage were collected over the course of the feeding experiment, freeze dried and ground though a 1.0 mm screen. Representative faecal samples were collected for three days (once a day) from each heifer as reported previously and washed (6-7 times) in 50 mM citrate buffer to remove solubles and to recover the final fiber residue. The material obtained after washing was termed total tract indigested fiber residue (TIFR), with the three samples being pooled. Barley silage was also washed through cheese cloth to obtain a similar particle size. Washed TIFR and barley silage were freeze dried and pretreated with alkaline peroxide using the procedure described earlier [9].

*(b) Enzymatic Fingerprinting of Alkaline Peroxide Treated Barley Silage and TIFR*. Enzymatic digestion of AP treated barley silage and TIFR was carried out in microassays as reported earlier [9]. Respective enzyme volumes containing defined protein contents for each reaction mixture were calculated according to statistical design detailed in Tables 2 and 3 and dispensed into a substrate slurry as described previously [9]. Samples were incubated at 50°C for 48 h on a rotating shaker at 10 rpm. After incubation, tubes were centrifuged at 1,500 ×g for 5 min and the supernatants (100 *μ*L) were heated at 90°C for 10 min to inactivate enzymes prior to determination of liberated glucose and xylose.

#### 2.3.2. Experiment Number 2: Effect of Enzymatic Prehydrolysis on Sugar Release from Alfalfa Hay and Barley Straw Exposed to Mixed Rumen Enzymes* In Vitro*

Enzyme prehydrolysis to enhance glucose yield from plant cell walls by mixed rumen enzymes was developed based on relative abundance data from the enzyme fingerprinting conducted in experiment 1 and differential Fourier transform infrared spectroscopy (FTIR) analysis of barley silage versus TIFR. The main objective of this experiment was to formulate mixed rumen enzymes in combination with recombinant enzymes in ratios that enhance plant cell wall digestion. Recombinant glycosyl hydrolases (GH) (endoglucanase GH7 (EGL7A_THITE)) and auxiliary enzymes, that is, esterase (AXE16A_ASPNG, AXE16B_ASPNG, FAE 1a), were used with barley straw, whereas hemicellulase polygalacturonase (PGA28A_ASPNG) and *α*-arabinofuranosidase (ABF54B_ASPNG) were used with alfalfa hay.

Alfalfa hay and barley straw were first ground to pass through a 1 mm screen and then were suspended separately at a final concentration of 0.5% in 50 mM sodium citrate (pH 5.0, containing 5 *μ*g/mL tetracycline, 5 *μ*g/mL cycloheximide, and 0.02% sodium azide). While the slurry was kept in suspension using a paddle reservoir designed for dispensing pharmaceutical beads (Biomek FXP, Model VP 756C-1P100, V&P Scientific, Inc., San Diego, CA), a total of 200 *μ*L (duplicate) of substrate slurry was dispensed into a mini-Eppendorf tube as described previously [9]. Defined protein content of each constituent enzyme for every reaction mixture (prehydrolysis) was calculated and dispensed according to the experimental design (Tables 3 and 4). Control samples were incubated without enzymes for 48 h at 50°C (total protein load 15 mg protein per g of glucan). After incubation, enzymes were inactivated by heating at 90°C for 15 min. Samples were allowed to cool and were subsequently centrifuged (1,500 ×g for 3 min) three times with 50 mM sodium citrate (pH 5.0, containing 5 *μ*g/mL tetracycline, 5 *μ*g/mL cycloheximide, and 0.02% sodium azide). Residues were added to mixed rumen enzymes at final concentration of 15 mg protein per g of glucan and incubated for further 48 h at 50°C. After incubation, the tubes were centrifuged at 1,500 ×g for 3 min to separate the solid residue from the supernatants (100 *μ*L) which were transferred into Costar 96-well plates and heated at 100°C for 10 min to inactivate enzymes.

### 2.4. Glucose and Xylose Assay, Scale-Up Assay, Total Glucan Content, and Attenuated Total Reflectance Fourier Transform Infrared Spectroscopy (ATR FT-IR) of Barley Silage and TIFR

Released glucose and xylose and total glucan contents of barley silage and TIFR were determined in a scale-up assay as previously reported [9]. ATR FT_IR analysis was also performed as documented earlier [12].

### 2.5. Data Analysis

Glucose and xylose released in each assay served as the response for experimental design in experiment 1, while in experiment 2 assay responses were expressed as a percentage yield of glucose in prehydrolyzed samples relative to the controls, where the residues were not prehydrolyzed. For experiments 1 and 2, ANOVA calculations were conducted and are reported in Tables 6 and 7, respectively.

## 3. Results and Discussions

**Figure 1:**
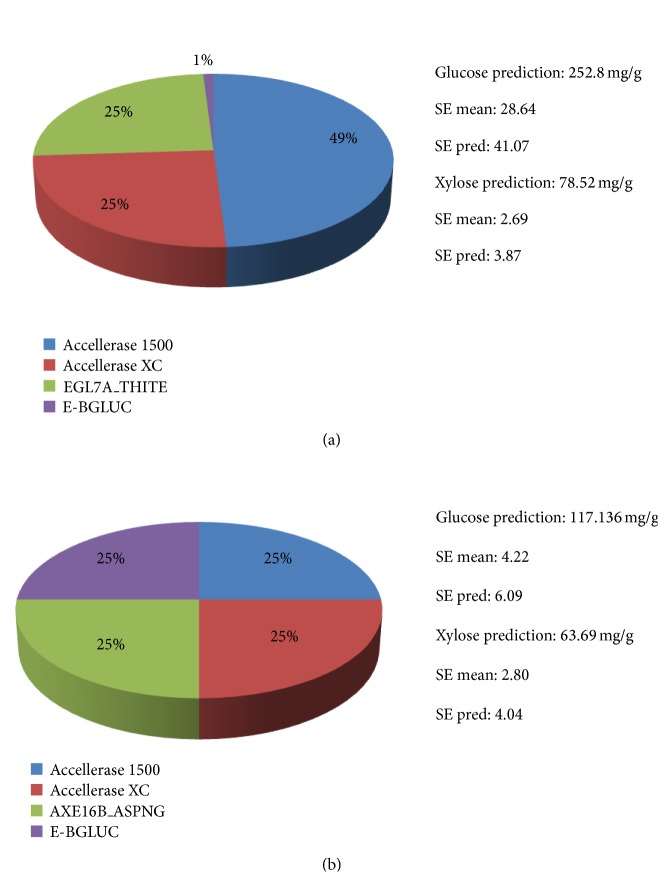
Enzyme fingerprinting of barley silage (a) and tract indigested fiber residues (TIFR) (b) for glucose and xylose released.

One increasingly important aspect of modern livestock production is the use of feed additives that aim to improve the efficiency of feed utilization and thereby contribute to the sustainability of meat and milk production. In monogastrics, exogenous enzymes have been used to remove antinutritional factors from feeds, to increase the digestibility of existing nutrients, and to complement the activity of endogenous enzymes [3, 4]. Digestion of plant cell walls to volatile fatty acids by ruminal microorganisms is a key step in the derivation of energy from recalcitrant substrates such as cereal straws by ruminants. Sufficient intake of digestible forage with an appropriate profile of nutrients is critical for optimal ruminant production. Hence, identification of those plant cell wall components that resist rumen digestion is vital for developing effective and efficient additives that improve the utilization of forages by ruminants. In this study, we used enzymatic fingerprinting of undigested fiber residue that has passed through the digestive tract (TIFR) to identify major undigested components of feed. We used two commercial enzymes (Accellerase 1500 and Accellerase XC) as core enzyme preparations as these two preparations are comprehensive and are routinely used for cell wall digestion. An enzyme cocktail containing 49% Accellerase 1500, 25% Accellerase XC, 25% of endoglucanase EGL7A_THITE, and 1% of *β*-glucosidase E-BGLUC activity resulted in the highest yield of glucose and xylose from AP treated barley silage (Figure 1(a)). Interestingly, enzyme fingerprinting of AP treated TIFR from cattle fed barley silage showed the highest sugar yield for the enzyme mix containing supplemental acetyl xylan esterase AXE16B_ASPNG (25%) and *β*-glucosidase E-BGLUC (25%) activity in addition to Accellerase 1500 (25%) and Accellerase XC (25%) (Figure 1(b)). These results suggest that effective digestion of AP treated TIFR increases with supplemental acetyl xylan esterase as well as *β*-glucosidase activity. With 22–50% of xylose residues being acetylated at the 0–2 and or 0–3 positions, acetylation has been reported to be an important factor influencing the digestibility of plant cell walls in ruminants [13]. In addition, arabinoxylan one of the main components in hemicellulose that forms the backbone structure of *β*-1, 4-linked xylose with arabinose side chains has been reported to be ester-linked to p-coumaric and ferulic acid and cross-linked to lignin via ferulic acid [14, 15].

Relatively lower yields were observed when the enzyme mix contained a higher percentage of core enzymes (only Accellerase 1500 and Accellerase XC, Tables 2 and 3). However, assays with high xylanase levels (Accellerase XC) produced higher glucose and xylose yield as compared to assays with high endoglucanase (Accellerase 1500) both from barley silage and TIFR (Tables 2 and 3). These results reflect the layered structure of cellulose and xylan chains within plant cell walls as xylan hydrolysis significantly improved the activity of cellulases against cellulose. Comparative analysis of results from enzymatic fingerprinting experiment for AP treated barley silage versus AP treated TIFR demonstrates that TIFR still contains a significant amount of residual sugars that could be released if digested with a suitable enzyme cocktail (Figure 1). Comprehensive saccharification of AP pretreated barley silage released 252 mg/g of glucose and 78 mg/g of xylose while 117 mg/g of glucose and 63 mg/g of xylose were released from AP TIFR. Thus, significant glucose (40%) and xylose (80%) were still recoverable from TIFR even after it had been subject to digestion within the intestinal tract of cattle (Figure 1). These results also suggest that cattle feces have considerable potential as a feedstock for biofuel production. Using manure as a feedstock for bioethanol production addresses some of the serious concerns raised against first generation biofuels in terms of their impact on biodiversity, competition for fuel versus food and carbon emissions.

**Figure 2:**
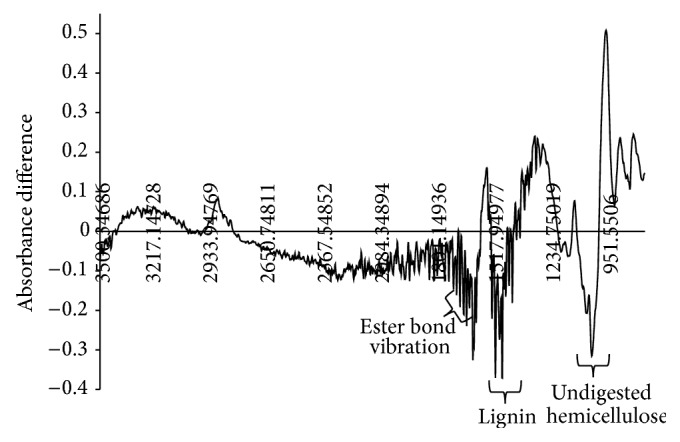
FTIR spectral difference for feed versus tract indigested fiber residues (TIFR) showing major undigested plant cell wall components after rumen digestion.

The high abundance of xylose in AP treated TIFR and critical requirement of acetyl xylan esterase reflects (Figure 1(b)) the recalcitrant nature of xylan and its cross-linked nature within the cell wall architecture. An abundance of undigested xylan and esterified hemicellulose components were also supported by differential spectra of barley silage versus TIFR by FTIR analysis. Peaks within the range of 1020 cm^−1^ to 1130 cm^−1^ corresponded to undigested arabinoglucuronoxylan, xyloglucan, arabinan, and pectin [16] and were reflective of an abundance of cross-linked hemicellulose within TIFR (Figure 2). Lignin was also concentrated in TIFR as indicated by spectral differences at 1508 cm^−1^ (aromatic skeletal vibration in lignin), 1541 cm^−1^ (C–H deformations; asymmetrical in –CH_3_ and –CH_2_), 1653 cm^−1^ (adsorbed O–H and conjugated C–O), and 1688 cm^−1^ (C=O in lignin) (Figure 2) [17]. Cross-linked esterified xylan and pectin in undigested residue were evident at peak 1714 cm^−1^ (C=O from xylan), 1738 cm^−1^ to 1747 cm^−1^ (unconjugated C=O stretch in xylan from acetic acid ester and pectin) [16, 17]. Our results are consistent with the notion that cross-linked xylan or ferulate-polysaccharide-lignin complexes are in part responsible for the recalcitrance of cellulose microfibrils [18, 19]. Similar results have been reported previously [9, 12] for barley straw where esterified pectin or xylan cross-linked to lignin was identified as the major factor responsible for the recalcitrance of these forages to mixed rumen enzymes as well as to commercial enzymes preparations.

We hypothesize that prehydrolysis of the forage with efficient auxiliary enzymes like esterases prior to consumption may increase fiber digestibility in ruminants by reducing recalcitrant cross-linked xylan content. This would be beneficial to ruminant production in the form of increased efficiency of meat and milk production.

**Figure 3:**
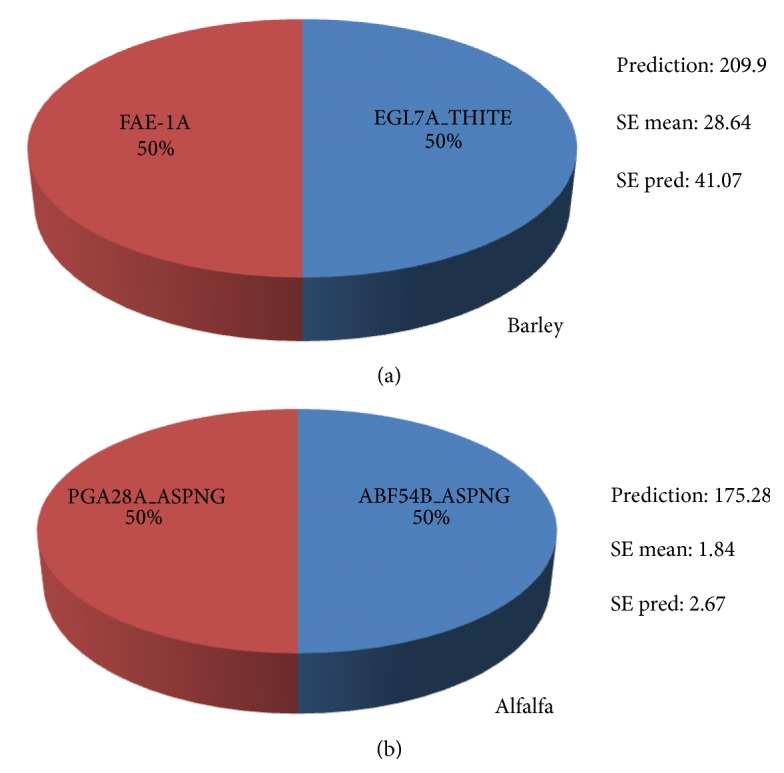
Optimization of enzyme ratios for enzyme prehydrolysis to aid high relative glucose yield from mixed rumen enzymes digestion of barley straw (a) and alfalfa hay (b).

Based on the results from the enzyme fingerprinting (experiment 1) we selected recombinant enzymes (namely acetyl xylan esterase AXE16A_ASPNG and AXE16B_ASPNG, polygalacturonase PGA28A_ASPNG, arabinofuranosidase ABF54B_ASPNG, and ferulic acid esterase FAE 1a) and endoglucanase EGL7A_THITE for prehydrolysis with an aim to increase the sugar yield from substrates exposed to mixed rumen enzymes. We specifically selected barley straw as the substrate in experiment 2 with the expectation that it would represent even a more recalcitrant forage source than barley silage. The model predicted a significant increase in glucose yield as result of enzymatic prehydrolysis of alfalfa hay and barley straw prior to digestion by mixed rumen enzymes (Figure 3). Prehydrolysis of barley straw with a mixture of endoglucanase GH 7 (EGL7A_THITE) and feruloyl esterase (FAE 1a: 1 : 1) prior to exposure to mixed rumen enzymes resulted in a 100% increase in glucose release as compared to the untreated control (Figure 3(a)), while for alfalfa hay, a 75% higher glucose yield was predicted by the model as a result of enzymatic prehydrolysis of alfalfa with a 1 : 1 ratio of polygalacturonase (PGA28A_ASPNG) and arabinofuranosidase (ABF54B_ASPNG) prior to digestion by mixed rumen enzymes (Figure 3(b)). These results are in agreement with major structural disparity between alfalfa and barley plant cell walls. The carbohydrates within barley plant cell walls are mainly cellulose and hemicellulose with a negligible amount of pectin [20], whereas alfalfa cell wall contains pectin and xylan in roughly similar proportions, each accounting for 15–20% of total cell wall carbohydrates [21]. Effectiveness of esterase (FAE 1a) as a prehydrolysis for barley straw digestion by mixed rumen enzymes is in accordance with earlier reports regarding esterified cross-linkages being the major factor limiting the hydrolysis of barley straw by rumen microbes [9]. However, the hydrolysis of hemicellulose in alfalfa by mixed rumen enzymes was enhanced by the addition of polygalacturonase (PGA28A_ASPNG) and arabinofuranosidase (ABF54B_ASPNG). These results suggested that multienzyme mixtures have potential as feed additives by initiating degradation of plant structural polysaccharides prior to ingestion by the ruminant animal.

**Figure 4:**
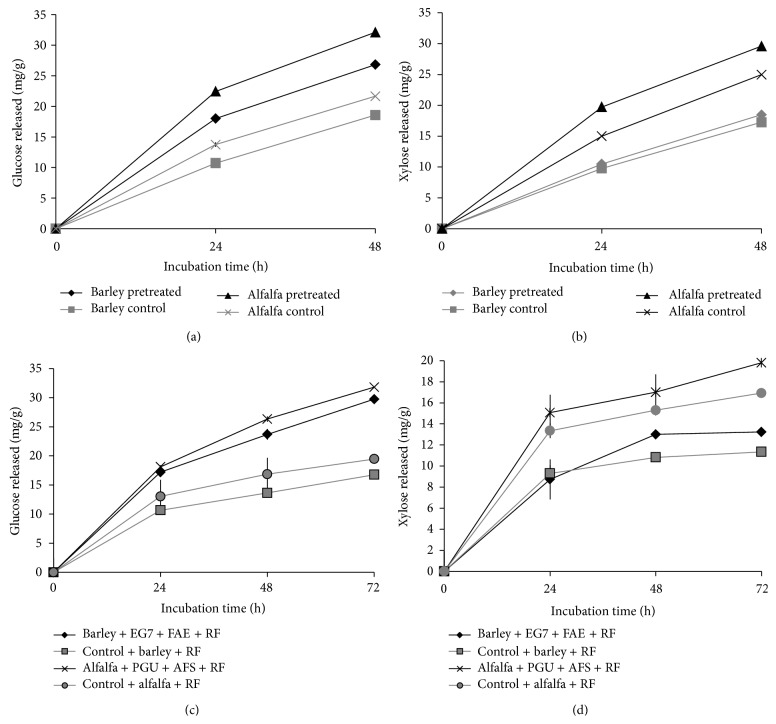
Glucose and xylose released as result of sequential ((a), (b)) and simultaneous ((c), (d)) hydrolysis of barley straw and alfalfa (biomass load: 2%, 30 mg of protein load/g of glucan in 5 mL reaction volume). Error bars (often invisible) represent SD of the mean (*n* = 8).

**Figure 5:**
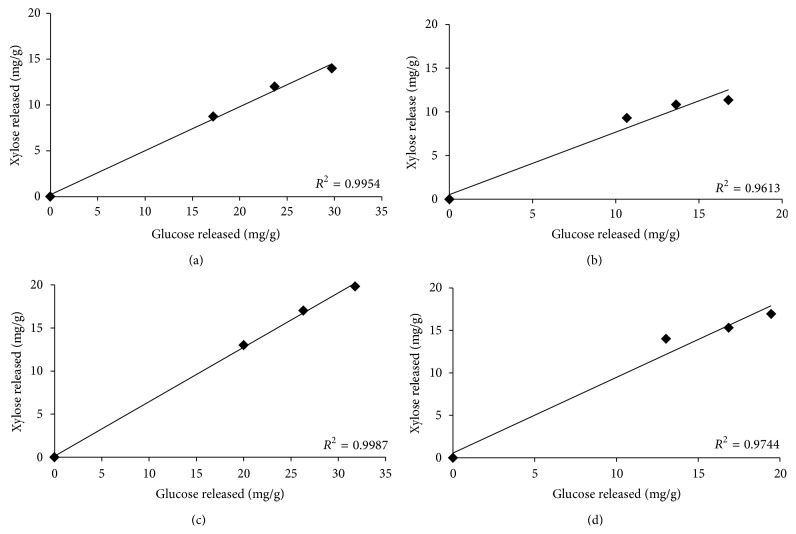
Change in glucan conversion plotted against change in xylan conversion for simultaneous hydrolysis of barley straw (a) alfalfa hay (c) by optimized enzyme mix and their respective controls ((b), (d); only rumen mix enzymes). Optimized enzyme mix composition used for barley straw and alfalfa hay digestion was identical to those used in Figure 3. Final enzymes load of 30 mg/g glucan with 2% solid load in 5 mL reaction volume was used.

Predictions made by our micromodel were also validated in scale-up assays that used a solid load of 2% w/v of barley straw or alfalfa hay. The effect of enzyme prehydrolysis on the subsequent enhancement of cell wall hydrolysis was studied by sequential or simultaneous addition of recombinant enzymes to mixed rumen enzymes for barley straw and alfalfa hay. Results of hydrolysis of barley straw and alfalfa hay by mixed rumen enzymes after 48 h of prehydrolysis by endoglucanase EGL7A_THITE (50%) and ferulic acid esterase FAE 1a (50%) for barley straw and polygalacturonase PGA28A_ASPNG (50%) and arabinofuranosidase ABF54B_ASPNG (50%) for alfalfa hay confirmed that these mixtures increased the release of glucose and xylose (*P* < 0.05) as a result of prehydrolysis (Figures 4(a) and 4(b)). Supplementation of rumen mixed enzymes with endoglucanase EGL7A_THITE (50%) and ferulic acid esterase FAE 1a (50%) during the digestion of barley straw and polygalacturonase PGA28A_ASPNG (50%) and arabinofuranosidase ABF54B_ASPNG (50%) with alfalfa hay enhanced (*P* < 0.05) digestion as compared to mixed rumen enzymes alone (Figures 4(c) and 4(d)). A direct relationship was observed between xylan conversion (fraction of available xylan converted) and glucose conversion (fraction of available glucan conversion) during the hydrolysis of plant cell walls (Figure 5). However, a stronger correlation between xylan and glucan digestion with added auxiliary enzymes for optimized mixed rumen enzymes (Figure 5) suggested improved glucan conversion, perhaps due to better xylan saccharification.

Comprehensive digestion of cell wall requires a battery of carbohydrases. Moreover, yields of recombinant enzymes from expression systems are often low. Hence, a microassay for screening novel enzymes against diverse biomass with ability to study synergy among hydrolases at low protein load is critical for development of enzyme formulations as additives to enhance ruminal digestion. In this study we successfully developed a microassay in combination with experimental design, to screen a number of recombinant enzymes at low protein loads, to enhance ruminal digestion of barley straw and alfalfa through augmentation of natural enzymatic activity in the rumen. Development of enzyme formulations that further enhance the utilization of low quality cellulosic feedstocks will ensure the sustainability of the beef industry in an environment of increasing demand for human food.

## 4. Conclusion

Enzyme fingerprinting was successfully used to identify principal recalcitrant constituent of barley silage. Cross-linked hemicelluloses as well as layered structure of cellulose and xylan were identified as prime recalcitrant factors to digestion. Partial digestion of hemicellulose in alfalfa hay and barley straw prior to ingestion with a cocktail of auxiliary enzymes significantly improved the hydrolysis of cellulose by mixed rumen enzymes. These results strengthen the rational of enzyme pretreatments targeting particular forage types. These same approaches could be used to improve the value of animal waste as a feedstock for biofuel production.

## Figures and Tables

**Table 1:** Sources of gene information, materials for production of recombinant enzymes, and properties of recombinant enzymes used in this study.

Clone ID	Entry name	Enzyme activity	CAZy family	Organism	mycoCLAP entry name	JGI ID	UniProt ID	Production host	pH optimum	Temp opt
Anig_Anig200605C	ABF54B_ASPNG	Alpha-N-arabinofuranosidase	GH54	*Aspergillus niger * N400	ABF54B_ASPNG	200605	P42255^a^	*Aspergillus niger* py11	4.5	50

Anig_Anig214598C	PGA28A_ASPNG	Endopolygalacturonase	GH28	*Aspergillus niger* N400	PGA28A_ASPNG	214598	Q9P4W4^a^	*Aspergillus niger* py11	4	60

Asn194096	AXE16B_ASPNG	Acetylesterase	CE16	*Aspergillus niger* N400	To be submitted	194096	G3Y7Z1^a^	*Aspergillus niger* py11	5	60

Asn7870	AXE16A_ASPNG	Acetylesterase	CE16	*Aspergillus niger* N400	To be submitted	54865	G3Y497^a^	*Aspergillus niger* py11	6	55

Anig_TterXXXX7G	EGL7A_THITE	Endoglucanase	GH7	*Thielavia terrestris *	To be submitted	54138	G2QZA7^b^	*Aspergillus niger* py11	6	55

—	FAE 1a	Ferulic acid esterase	CE1	*Anaeromyces mucronatus *	—	—	F2YCB6^c^	*E. coli *	7.2	37

^a^The ID number corresponds to the protein ID of *Aspergillus niger* v3.0 annotation, http://genome.jgi-psf.org/Aspni5; ^b^the ID number corresponds to the protein ID of *Thielavia terrestris* v3.0 annotation, http://genome.jgi-psf.org/Thite2. ^c^ID for FAE 1a is a primary (citable) accession number (http://www.uniprot.org/uniprot/F2YCB6).

**Table 2:** Experimental design for comprehensive digestion of barley silage.

Std	Run	Component 1	Component 2	Component 3	Component 4	Component 5	Component 6	Response 1	Response 2
		A: acell 1500	B: acell XC	C: E-BGLUC	D: FAE	E: AXE16A_ASPNG	F: EGL7A_THITE	Glucose rel	Xylose rel
12	1	0.250	0.500	0.250	0.000	0.000	0.000	153.436	36.7534
4	2	0.250	0.250	0.000	0.500	0.000	0.000	166.702	30.6087
10	3	0.500	0.250	0.000	0.000	0.250	0.000	172.536	50.8231
24	4	0.292	0.292	0.292	0.042	0.042	0.042	182.126	48.4112
3	5	0.250	0.250	0.500	0.000	0.000	0.000	135.056	32.2167
20	6	0.250	0.250	0.000	0.250	0.000	0.250	190.597	52
1	7	0.750	0.250	0.000	0.000	0.000	0.000	152.397	39.9119
28	8	0.333	0.333	0.083	0.083	0.083	0.083	177	54
18	9	0.250	0.250	0.250	0.000	0.000	0.250	203.223	69.6018
15	10	0.250	0.500	0.000	0.000	0.000	0.250	178.849	65.2948
8	11	0.500	0.250	0.250	0.000	0.000	0.000	135.456	37.5574
11	12	0.500	0.250	0.000	0.000	0.000	0.250	251.492	78.2159
25	13	0.292	0.292	0.042	0.292	0.042	0.042	172.776	44.9655
14	14	0.250	0.500	0.000	0.000	0.250	0.000	175.093	57.3124
13	15	0.250	0.500	0.000	0.250	0.000	0.000	179.968	49.4449
17	16	0.250	0.250	0.250	0.000	0.250	0.000	156.313	58.2887
9	17	0.500	0.250	0.000	0.250	0.000	0.000	169.659	44.1616
22	18	0.542	0.292	0.042	0.042	0.042	0.042	209.776	52.2014
26	19	0.292	0.292	0.042	0.042	0.292	0.042	190	63.6294
23	20	0.292	0.542	0.042	0.042	0.042	0.042	190	52
7	21	0.500	0.500	0.000	0.000	0.000	0.000	215.61	50
21	22	0.250	0.250	0.000	0.000	0.250	0.250	182.605	53.6945
27	23	0.292	0.292	0.042	0.042	0.042	0.292	200	62
6	24	0.250	0.250	0.000	0.000	0.000	0.500	198.828	65.6968
5	25	0.250	0.250	0.000	0.000	0.500	0.000	215.21	77.9288
16	26	0.250	0.250	0.250	0.250	0.000	0.000	182.845	37.5
2	27	0.250	0.750	0.000	0.000	0.000	0.000	180.208	61.16
19	28	0.250	0.250	0.000	0.250	0.250	0.000	163.506	49.6172

**Table 3:** Experimental design for comprehensive digestion of TIFR.

Std	Run	Component 1	Component 2	Component 3	Component 4	Component 5	Component 6	Response 1	Response 2
		A: acell 1500	B: acell XC	C: E-BGLUC	D: FAE	E: AXE16A_ASPNG	F: EGL7A_THITE	Glucose rel	Xylose rel
12	1	0.250	0.500	0.250	0.000	0.000	0.000	65.7698	36.3515
4	2	0.250	0.250	0.000	0.500	0.000	0.000	64.731	27.1631
10	3	0.500	0.250	0.000	0.000	0.250	0.000	90.6233	59.6095
24	4	0.292	0.292	0.292	0.042	0.042	0.042	91.8221	44.6784
3	5	0.250	0.250	0.500	0.000	0.000	0.000	78.6361	31.585
20	6	0.250	0.250	0.000	0.250	0.000	0.250	87.5866	49.5597
1	7	0.750	0.250	0.000	0.000	0.000	0.000	84.0703	42.3813
28	8	0.333	0.333	0.083	0.083	0.083	0.083	87	47.0904
18	9	0.250	0.250	0.250	0.000	0.000	0.250	89.8242	54.4985
15	10	0.250	0.500	0.000	0.000	0.000	0.250	94.2994	53.6371
8	11	0.500	0.250	0.250	0.000	0.000	0.000	85.9084	37.0406
11	12	0.500	0.250	0.000	0.000	0.000	0.250	100.053	56.2213
25	13	0.292	0.292	0.042	0.292	0.042	0.042	90.4635	40
14	14	0.250	0.500	0.000	0.000	0.250	0.000	77.0378	56.6807
13	15	0.250	0.500	0.000	0.250	0.000	0.000	95.7379	41.9219
17	16	0.250	0.250	0.250	0.000	0.250	0.000	117.315	63.1126
9	17	0.500	0.250	0.000	0.250	0.000	0.000	90.5434	35.2029
22	18	0.542	0.292	0.042	0.042	0.042	0.042	93	52.7182
26	19	0.292	0.292	0.042	0.042	0.292	0.042	84.39	58.5758
23	20	0.292	0.542	0.042	0.042	0.042	0.042	90.7032	53.2351
7	21	0.500	0.500	0.000	0.000	0.000	0.000	86.9473	44.3338
21	22	0.250	0.250	0.000	0.000	0.250	0.250	82.7917	50.536
27	23	0.292	0.292	0.042	0.042	0.042	0.292	85.7485	53
6	24	0.250	0.250	0.000	0.000	0.000	0.500	98.8546	59.6095
5	25	0.250	0.250	0.000	0.000	0.500	0.000	60.2557	60
16	26	0.250	0.250	0.250	0.250	0.000	0.000	64.3314	31.8147
2	27	0.250	0.750	0.000	0.000	0.000	0.000	93.2605	46.6884
19	28	0.250	0.250	0.000	0.250	0.250	0.000	84.7096	48.2389

**Table 4:** Experimental design for comprehensive digestion of alfalfa hay by rumen mix enzymes after 48 h of enzyme prehydrolysis.

Std	Run	Component 1	Component 2	Component 3	Component 4	Component 5	Component 6	Component 7	Response 1
		A: EGL7A_THITE	B: AXE16A_ASPNG	C: ASN ACEA	D: AXE16B_ASPNG	E: FAE	F: ABF54B_ASPNG	G: PGA28A_ASPNG	Glucose release %
10	1	50.000	0.000	0.000	50.000	0.000	0.000	0.000	145
24	2	0.000	0.000	0.000	50.000	0.000	50.000	0.000	149
19	3	0.000	0.000	50.000	50.000	0.000	0.000	0.000	134
12	4	50.000	0.000	0.000	0.000	0.000	50.000	0.000	144
41	5	0.000	0.000	0.000	0.000	100.000	0.000	0.000	108
40	6	0.000	0.000	0.000	100.000	0.000	0.000	0.000	135
29	7	57.143	7.143	7.143	7.143	7.143	7.143	7.143	153
9	8	50.000	0.000	50.000	0.000	0.000	0.000	0.000	166
7	9	0.000	0.000	0.000	0.000	0.000	0.000	100.000	141
26	10	0.000	0.000	0.000	0.000	50.000	50.000	0.000	126
32	11	7.143	7.143	7.143	57.143	7.143	7.143	7.143	143
6	12	0.000	0.000	0.000	0.000	0.000	100.000	0.000	141
28	13	0.000	0.000	0.000	0.000	0.000	50.000	50.000	176
13	14	50.000	0.000	0.000	0.000	0.000	0.000	50.000	143
22	15	0.000	0.000	50.000	0.000	0.000	0.000	50.000	139
31	16	7.143	7.143	57.143	7.143	7.143	7.143	7.143	145
15	17	0.000	50.000	0.000	50.000	0.000	0.000	0.000	145
36	18	14.286	14.286	14.286	14.286	14.286	14.286	14.286	148
37	19	100.000	0.000	0.000	0.000	0.000	0.000	0.000	148
8	20	50.000	50.000	0.000	0.000	0.000	0.000	0.000	160
27	21	0.000	0.000	0.000	0.000	50.000	0.000	50.000	149
21	22	0.000	0.000	50.000	0.000	0.000	50.000	0.000	137
11	23	50.000	0.000	0.000	0.000	50.000	0.000	0.000	131
35	24	7.143	7.143	7.143	7.143	7.143	7.143	57.143	146
33	25	7.143	7.143	7.143	7.143	57.143	7.143	7.143	136
38	26	0.000	100.000	0.000	0.000	0.000	0.000	0.000	131
30	27	7.143	57.143	7.143	7.143	7.143	7.143	7.143	139
17	28	0.000	50.000	0.000	0.000	0.000	50.000	0.000	141
5	29	0.000	0.000	0.000	0.000	100.000	0.000	0.000	109
2	30	0.000	100.000	0.000	0.000	0.000	0.000	0.000	134
23	31	0.000	0.000	0.000	50.000	50.000	0.000	0.000	132
16	32	0.000	50.000	0.000	0.000	50.000	0.000	0.000	128
4	33	0.000	0.000	0.000	100.000	0.000	0.000	0.000	
3	34	0.000	0.000	100.000	0.000	0.000	0.000	0.000	138
20	35	0.000	0.000	50.000	0.000	50.000	0.000	0.000	154
14	36	0.000	50.000	50.000	0.000	0.000	0.000	0.000	129
34	37	7.143	7.143	7.143	7.143	7.143	57.143	7.143	147
25	38	0.000	0.000	0.000	50.000	0.000	0.000	50.000	139
18	39	0.000	50.000	0.000	0.000	0.000	0.000	50.000	138
39	40	0.000	0.000	100.000	0.000	0.000	0.000	0.000	138
1	41	100.000	0.000	0.000	0.000	0.000	0.000	0.000	153

**Table 5:** Experimental design for comprehensive digestion of barley straw by rumen mix enzymes after 48 h of enzyme prehydrolysis.

Std	Run	Component 1	Component 2	Component 3	Component 4	Component 5	Component 6	Component 7	Response 1
		A: EGL7A_THITE	B: AXE16A_ASPNG	C: ASN ACEA	D: AXE16B_ASPNG	E: FAE	F: ABF54B_ASPNG	G: PGA28A_ASPNG	Glucose release %
14	1	0.000	0.500	0.500	0.000	0.000	0.000	0.000	158
31	2	0.071	0.071	0.571	0.071	0.071	0.071	0.071	139
18	3	0.000	0.500	0.000	0.000	0.000	0.000	0.500	268
19	4	0.000	0.000	0.500	0.500	0.000	0.000	0.000	
36	5	0.143	0.143	0.143	0.143	0.143	0.143	0.143	160
7	6	0.000	0.000	0.000	0.000	0.000	0.000	1.000	139
12	7	0.500	0.000	0.000	0.000	0.000	0.500	0.000	122
39	8	0.000	0.000	1.000	0.000	0.000	0.000	0.000	270
21	9	0.000	0.000	0.500	0.000	0.000	0.500	0.000	89
2	10	0.000	1.000	0.000	0.000	0.000	0.000	0.000	268
40	11	0.000	0.000	0.000	1.000	0.000	0.000	0.000	183
32	12	0.071	0.071	0.071	0.571	0.071	0.071	0.071	154
34	13	0.071	0.071	0.071	0.071	0.071	0.571	0.071	162
26	14	0.000	0.000	0.000	0.000	0.500	0.500	0.000	131
6	15	0.000	0.000	0.000	0.000	0.000	1.000	0.000	95
1	16	1.000	0.000	0.000	0.000	0.000	0.000	0.000	249
24	17	0.000	0.000	0.000	0.500	0.000	0.500	0.000	181
8	18	0.500	0.500	0.000	0.000	0.000	0.000	0.000	246
17	19	0.000	0.500	0.000	0.000	0.000	0.500	0.000	203
35	20	0.071	0.071	0.071	0.071	0.071	0.071	0.571	
41	21	0.000	0.000	0.000	0.000	1.000	0.000	0.000	203
27	22	0.000	0.000	0.000	0.000	0.500	0.000	0.500	207
9	23	0.500	0.000	0.500	0.000	0.000	0.000	0.000	297
4	24	0.000	0.000	0.000	1.000	0.000	0.000	0.000	230
10	25	0.500	0.000	0.000	0.500	0.000	0.000	0.000	206
23	26	0.000	0.000	0.000	0.500	0.500	0.000	0.000	191
15	27	0.000	0.500	0.000	0.500	0.000	0.000	0.000	243
20	28	0.000	0.000	0.500	0.000	0.500	0.000	0.000	305
33	29	0.071	0.071	0.071	0.071	0.571	0.071	0.071	236
28	30	0.000	0.000	0.000	0.000	0.000	0.500	0.500	337
29	31	0.571	0.071	0.071	0.071	0.071	0.071	0.071	254
30	32	0.071	0.571	0.071	0.071	0.071	0.071	0.071	247
5	33	0.000	0.000	0.000	0.000	1.000	0.000	0.000	235
3	34	0.000	0.000	1.000	0.000	0.000	0.000	0.000	341
37	35	1.000	0.000	0.000	0.000	0.000	0.000	0.000	262
11	36	0.500	0.000	0.000	0.000	0.500	0.000	0.000	370
16	37	0.000	0.500	0.000	0.000	0.500	0.000	0.000	296
13	38	0.500	0.000	0.000	0.000	0.000	0.000	0.500	214
22	39	0.000	0.000	0.500	0.000	0.000	0.000	0.500	271
25	40	0.000	0.000	0.000	0.500	0.000	0.000	0.500	276
38	41	0.000	1.000	0.000	0.000	0.000	0.000	0.000	375

**Table 6:** ANOVA calculations of *F*-value, *P* value, *R* ^2^, adjusted *R* ^2^, predicted *R* ^2^, and adequate precision as calculated by the Design-Expert software for enzymatic fingerprinting of barley silage and tract indigested fiber residue (TIFR).

Source	Enzyme source	*F*-value	*P* value	*R*-square	Adjusted *R*-square	Predicted *R*-square	Difference between Adj and Pred *R*-square	Adequate precision
AP pretreated barley silage feed	Accell1500 + AccellXC + EGL7A_THITE + E-BGLUC	129.1	<0.0001	0.98	0.97	0.86	0.11	52.3

AP pretreated TIFR	Accell1500 + AccellXC + AXE16B_ASPNG + E-BGLUC	16.60	<0.0001	0.88	0.83	0.71	0.12	16.8

**Table 7:** ANOVA calculations of *F*-value, *P* value, *R* ^2^, adjusted *R* ^2^, predicted *R* ^2^, and adequate precision as calculated by the Design-Expert software for enzymatic prehydrolysis of alfalfa hay and barley straw on final saccharification yield from rumen mix enzyme digestion.

Feedstock	Enzyme source	*F*-value	*P* value	*R*-square	Adjusted *R*-square	Predicted *R*-square	Difference between Adj and Pred *R*-square	Adequate precision
Alfalfa	Enzyme pretreatments followed by rumen enzyme mix	129.1	<0.0001	0.98	0.97	0.86	0.11	52.3

Barley	Enzyme pretreatments followed by rumen enzyme mix	16.60	<0.0001	0.88	0.83	0.71	0.12	16.8
